# Extracellular vesicles of *Janthinobacterium lividum* as violacein carriers in melanoma cell treatment

**DOI:** 10.1007/s00253-024-13358-1

**Published:** 2024-12-05

**Authors:** Patrycja Kowalska, Jolanta Mierzejewska, Paulina Skrzeszewska, Aleksandra Witkowska, Katarzyna Oksejuk, Ewa Sitkiewicz, Mariusz Krawczyk, Magdalena Świadek, Agata Głuchowska, Klaudia Marlicka, Anna Sobiepanek, Małgorzata Milner-Krawczyk

**Affiliations:** 1 Chair of Drug and Cosmetics Biotechnology, Warsaw University of Technology, Warsaw, Poland; 2 Doctoral School Warsaw University of Technology, Warsaw, Poland; 3 Mass Spectrometry Laboratory, Institute of Biochemistry and Biophysics, Polish Academy of Science, Warsaw, Poland; 4 Genomed S.A., Warsaw, Poland; 5 Nencki Institute of Experimental Biology, Polish Academy of Science, Warsaw, Poland

**Keywords:** Violacein, Extracellular vesicles, EVs, *Janthinobacterium lividum*, Drug carriers, Melanoma

## Abstract

**Abstract:**

Violacein is a natural indole-derived purple pigment of microbial origin that has attracted attention for its remarkable biological properties. Due to its poor solubility in aqueous media, most studies of this pigment use extracts of the compound obtained with common solvents. Violacein is also transported in bacterial extracellular vesicles (EVs) and transferred via this type of carrier remains stable in an aqueous environment. This paper is the first to present an in-depth study of *Janthinobacterium lividum* EVs as violacein carriers. *J. lividum* EVs were studied for their contribution to violacein translocation, size, morphology and protein composition. The production of violacein encapsulated in EVs was more efficient than the intracellular production of this compound. The average size of the violacein-containing EVs was 124.07 ± 3.74 nm. Liquid chromatography-tandem mass spectrometry analysis (LC–MS/MS) revealed 932 proteins common to three independent EVs isolations. The high proportion of proteins with intracellular localisation, which are involved in many fundamental cellular processes, suggests that *J. lividum* EVs could be generated in a cell lysis model, additionally stimulated by violacein production. Using human keratinocytes and melanoma cell lines, it was confirmed that *J. lividum* EVs are able to react with and deliver their cargo to mammalian cells. The EVs-delivered violacein was shown to retain its activity against melanoma cells, and the dose and timing of treatment can be selected to target only cancer cells. The characterisation of *J. lividum* EVs, described in the following paper, represents a milestone for their future potential anticancer application.

**Key points:**

• *This report focuses on the investigation of Janthinobacterium lividum EVs as a new delivery vehicle for violacein, a compound with a previously demonstrated broad spectrum of activity.*

• *EVs were characterised for size, morphology and protein composition.*

• *Studies on human keratinocytes and a melanoma cell model confirmed that the activity of violacein applied in the encapsulated form of EVs is similar to that of its organic solvent extract, but their production is much more environmentally friendly.*

**Supplementary Information:**

The online version contains supplementary material available at 10.1007/s00253-024-13358-1.

## Introduction

Violacein is a natural, indole-derived, purple pigment that has attracted attention for its remarkable biological and physical properties. It is produced by at least 11 known bacterial genera, including *Chromobacterium*, *Janthinobacterium*, *Iodobacter*, *Duganella*, *Collimonas*, *Pseudoalteromonas*, *Massilia*, *Pseudoduganella*, *Archangium*, *Microbulbifer* and *Chitinimonas* (Choi et al. 2015; De León et al. 2023). Because of its physiological and environmental role, this compound has attracted interest primarily as a potential drug against major human pathogens (Ahmed et al. 2021), especially as it has been found to act synergistically with many commercial antibiotics and could be used as a drug in combination with other antimicrobial agents (Subramaniam et al. 2014). Moreover, its activity against main skin pathogens e.g. the Gram-positive bacterium *Staphylococcus aureus* and the fungi *Trichophyton rubrum* and *Candida albicans*, has been proven (Sasidharan et al. 2015; Kanelli et al. 2018; Cauz et al. 2019). Over time, however, the role that violacein can play in fighting cancer cells has been shown to be equally important. In this regard, many studies have been published on both the effect of this compound and the mechanism of its action on a number of cancer cell lines such as lung cancer cells (Melo et al. 2000), colorectal adenoma cells (Kodach et al. 2006), acute myeloid leukaemia cells (Durán et al. 2016), or melanoma cells (Mojib et al. 2011; Gonçalves et al. 2016; Aires-Lopes et al. 2023). Interestingly, a significant difference in the response of cancerous and noncancerous cells to violacein was observed in a mouse cell model (Mojib et al. 2011), further highlighting the therapeutic potential of this compound. In addition, Gonçalves et al. (2016) observed that violacein reduces the invasive potential of melanoma cells and has a much more potent anticancer effect than temozolomide, a drug used in the standard treatment of this skin cancer. Similarly, Aires-Lopes et al. (2023) published results showing that violacein synergistically improves the response to vemurafenib in melanoma spheroids. The above facts and discoveries indicate the high potential of using violacein as a therapeutic and protective substance for topical application to the skin, although further research in this direction should be undertaken. Considering the potential applications of violacein, the challenge is to develop a strategy to overcome its hydrophobicity (log *P*_octanol:water_ = 3.34), which makes this compound unstable in the aquatic environment, that is natural for all living organisms (Choi et al. 2020). As previously described, violacein is well soluble in alcohols (such as methanol or ethanol), dimethyl sulfoxide or acetone (Pantanella et al. 2007; Masuelli et al. 2016; Durán et al. 2021). Consequently, the vast majority of research on this compound has been carried out using its more or less purified cellular extracts prepared in solvents. So far, several types of violacein stabilisers have been developed (Arif et al. 2017; Berti et al. 2019; Nakazato et al. 2019; Durán et al. 2021; Hamzah et al. 2024), but often the best and simplest solutions are provided by nature itself. In 2020, it was reported that violacein could remain stable in an aqueous environment when safely enclosed in the extracellular vesicles (EVs) of *Chromobacterium violaceum* (Choi et al. 2020) which increased its solubility by 1740-fold. In the same year, another publication on violacein-containing EVs (Batista et al. 2020) demonstrated that the outer membrane vesicles produced by *C. violaceum* deliver violacein to mediate its antimicrobial toxicity over long distances. However, no research has been presented on the potential anticancer use of EVs-containing violacein.

The violacein-containing EVs used in this work are from a strain assigned to the genus *Janthinobacterium*. The most common characteristics of the genus *Janthinobacterium* are Gram-negative, rod-shaped, aerobic bacteria that are usually found in various environments, including soil, waterways, food and the skin of vertebrates, including humans (Ramsey et al. 2015). Whilst members of the *Janthinobacterium* sp. appear to be non-pathogenic to humans, animals and plants, they are known to have a strong impact on serious human pathogens, both fungal and bacterial (Haack et al. 2016; Baricz et al. 2018). Furthermore, as a permanent component of the human microflora (Grice et al. 2008; Yang et al. 2022), bacteria of this genus have been suggested as excellent candidates for probiotic use (Ramsey et al. 2015). There are only two reports that slightly disturb the positive picture of this bacteria genus: an isolated case of septicemia in humans (Patijanasoontorn et al. 1992) and the report that the genus *Janthinobacterium* was a little more abundant in the blood microbiome of patients with major depression (Cheng et al. 2023).

EVs-based intercellular communication is conserved throughout the living world and across kingdoms. Many studies suggest that these structures have several advantages over conventional synthetic nanocarriers, including their low immunogenicity or good biocompatibility, so they can serve as natural carriers for therapeutic agents and drugs (Herrmann et al. 2021; Du et al. 2023). However, the possibility of using a particular type of vesicles must be supported by detailed research into its composition to rule out any side effects. This is especially true for EVs of microbial origin, which are often described as carriers of virulence factors. In this work, we used nanoparticle tracking analysis, electron microscopy imaging and mass spectrometry analysis to characterise EVs from *Janthinobacterium lividum* in terms of size, morphology and protein composition. We have also shown that EV-delivered violacein affects the actin cytoskeleton and induces apoptosis in melanoma cells, and that low doses of violacein selectively reduce the growth of human cancer cells.

## Methods

### Bioproduction of violacein

Crude methanol extract of violacein (Ex-Vio) and vesicles containing violacein (EVs-Vio) were obtained from a production culture of the *J. lividum* PCM 3520 strain isolated from water samples taken from a deep well in Poland (Supplemental Table S1, Fig. S1). The strain was deposited at the Polish Collection of Microorganisms. The starter culture was obtained by inoculating 20 ml of ½ LB liquid medium (LB broth; BioShop, Burlington, Canada) in a 300-ml flask with a single colony taken from the ½ LB agar medium (LB agar; BioShop, Burlington, Canada) and carried out on a shaker at 110 rpm at 20 °C for 48 h. After this time, glycerol was added to the culture to the final concentration of 17% (v/v); then, the culture was aliquoted (1.5 ml) and frozen at − 80 °C. The production culture was started by inoculating 100 ml of ½ LB liquid medium in a 500-ml flask with a thawed inoculum (1.5 ml). Cultivation was carried out on a shaker at 110 rpm at 20 °C. After 5 days, the culture was centrifuged (15 min, 4 °C, 47808 × *g*) to obtain two fractions: the supernatant with vesicles containing violacein (EVs-Vio) and the cell pellet. The latter one was frozen at − 20 °C for subsequent extraction of violacein.

The purification procedure for EVs-Vio was based on the use of filters with different cut-off points as described recently (Mierzejewska et al. 2023). Briefly, the supernatant was first passed through a 0.22-μm filter to remove residual bacterial cells and cell debris (Bottle-top Vacuum Filtration Systems, SFCA, VWR, Radnor, PA, USA). The EVs-Vio present in the permeate were then concentrated on a 100 kDa filter (Amicon Ultra-15 MWCO 100 kDa, Merck, Darmstadt, Germany) using centrifugation cycles of 20 min, 4900 × *g* and 4 °C. It should be noted that the filtrates were straw yellow in colour and that 343.34 Da violacein was completely absent from this fraction (Supplemental Fig. S1-S2). Finally, the obtained EVs-Vio’s concentrate was washed three times with phosphate-buffered saline (PBS) (at centrifugation cycles of 20 min, 4900 × *g* and 4 °C). The resulting samples of EVs-Vio were aliquoted and stored at − 80 °C.

The thawed pellet of bacterial cells harvested from 50 ml of production culture was mixed with 20 ml of pure methanol (MilliporeSigma, Burlington, VT, USA) and shaken for 30 min at 20 °C (150 rpm). It was then centrifuged (15 min, 4 °C, 47808 × *g*) and the supernatant was transferred to a round bottom flask. Subsequently, the methanol was evaporated from the supernatant (if necessary, the remaining residual water was removed using a vacuum pump). The remaining violacein precipitate in the flask was redissolved in pure methanol, centrifuged (5 min, 15700 × *g,* room temperature), aliquoted and stored at − 80 °C. The quality of the violacein extract (Ex-Vio) was checked each time by high-performance liquid chromatography (HPLC) analysis (Supplemental Fig. S3).

The concentration of violacein in the extract and in the vesicles was determined from measured absorbance values at 577 nm of samples diluted at least 20-fold in pure methanol. The corresponding calculations were made using the Beer-Lambert law and the extinction constant (ε = 1.7 × 10^4^ l mol^−1^ cm^−1^) (Antônio and Creczynski-Pasa 2004). The formula used to perform the calculations was $c=A/(\varepsilon \times l)$ where *A* is the absorbance at 577 nm, *c* is the molar concentration of the compound, *ε* is the violacein molar extinction coefficient, and $l$ is the thickness of the absorbing layer.

### Measurement of violacein bioproduction efficiency

The production efficiency of violacein in its intracellular form and encapsulated in EVs was monitored by daily analysis of 1 ml samples taken from the production culture. In order to separate the bacterial cells from the medium containing EVs, the samples were centrifuged at 13000 × *g* for 5 min at room temperature (RT). To measure the concentration of violacein encapsulated in the form of EVs, the resulting supernatant was diluted in pure methanol (at least × 50). Subsequently, crude violacein was extracted from the cell sediments with 1 ml of methanol after incubation for 30 min at 30 °C with vortex shaking. The violacein extract was then centrifuged (5 min, 15700 × *g*, 4 °C) and the amount of violacein in the supernatant was checked according to the Beer‒Lambert law, as mentioned above.

### Nanoparticle tracking analysis (NTA) of EVs-Vio

The size distribution and concentration of EVs in the sample were measured using a NanoSight Pro (Malvern Panalytical Ltd., Malvern, UK) equipped with a 488 nm blue laser and a 500 nm detector. Each measurement was performed in 10 independent videos under the following parameters: diluent, water; temperature, 24.0–25.1 °C; viscosity, 0.9100–0.8869 cP; pump speed, 2.5 μL/min; focus position, 3280–3300; exposure time, 29.5–31.2 ms; contrast gain, 4.5–5.5; display brightness, 3; and with light scattering filter. Videos were generated from 750 frames. Prior to measurement, each sample was diluted in 1 × PBS (filtered through a 0.1 μm filter) to estimate 50–80 EVs per frame of camera detection (dilution factor was between × 1000 and × 16000). Raw data were analysed using the built-in software NS XPLORER v 1.1.0.6 under the FTLA (finite track length adjustment) distribution. Final result averaged from measurements of 9 independent EVs isolations.

### Transmission electron microscopy

The morphology of *J. lividum* EVs samples was analysed by the transmission electron microscopy (TEM) at the Laboratory of Electron Microscopy, which serves as an imaging core facility at the Nencki Institute of Experimental Biology Polish Academy of Science and is part of the infrastructure of the Polish Euro-BioImaging Node. A sample of bacterial extracellular vesicles was placed on a Formvar/carbon-coated copper grid (200 mesh, Ted Pella Inc., Redding, CA, USA) and incubated at room temperature for 20 min. After incubation, the grid was dried with tissue paper and a 1% (w/v) glutaraldehyde (Electron Microscopy Sciences, Hatfield, PA, USA) solution in PBS was applied for 5 min to fix the sample. The grid was then washed with distilled water, 10 times for 1 min each wash. After rinsing off the fixative, the grids were stained with a 2% (w/v) aqueous uranyl acetate solution (Serva, Heidelberg, Germany) and incubated in the dark for 5 min. Excess uranyl acetate was removed with tissue paper. The grids were then dried at room temperature for 24 h and examined using a JEM 1400 (JEOL Ltd., Tokyo, Japan) transmission electron microscope.

### Isolation of proteins from EVs

Freshly isolated EVs suspension (400 µl) was mixed with 100% (v/v) trichloroacetic acid (TCA; MilliporeSigma, Burlington, VT, USA) and 0.15% (v/v) sodium deoxycholate (DOC; MilliporeSigma, Burlington, VT, USA) in a ratio of 4:1. Homogenisation of the samples was performed by intensive vortexing for 10 min at room temperature. The sample was then incubated for 30 min at room temperature and further centrifuged (15700 × *g*, 15 min, 4 °C). The supernatant was discarded and the pellet was rinsed three times with 100% acetone and then dried for several minutes at 37 °C. The resulting pellets were resuspended in 40 μl of onefold concentrated protein loading buffer (EURx, Gdansk, Poland), denatured and analysed by sodium dodecyl-sulphate polyacrylamide gel electrophoresis (SDS-PAGE) conducted according to a standard protocol (Gallagher 2012).

### Identification of proteins isolated from EVs of *J. lividum* by mass spectrometry and bioinformatics analysis

The EVs proteins were analysed by the liquid chromatography-tandem mass spectrometry analysis (LC–MS/MS) at the Mass Spectrometry Laboratory of the Institute of Biochemistry and Biophysics of the Polish Academy of Sciences. The protein precipitates were resuspended in 50 µl of 20% 2,2,2-trifluoroethanol in 100 mM ammonium bicarbonate and the basic steps of the analysis were carried out as previously described (Mierzejewska et al. 2023). After protein digestion with trypsin, the next step was peptide purification using a single-pot solid-phase-enhanced sample preparation (SP3). Magnetic bead mixtures were prepared by combining equal amounts of Sera-Mag Carboxyl hydrophilic and hydrophobic particles (09–981-121 and 09–981-123, GE Healthcare, Chicago, IL, USA). The bead mixture was washed three times with mass spectrometry (MS) grade water and resuspended to a working concentration of 10 µg/µl. The bead mixture was then added to samples suspended in 100% acetonitrile (MilliporeSigma, Burlington, VT, USA); this step was repeated twice. Pure peptides were eluted from the beads using 2% acetonitrile in MS grade water. A magnet was used to separate the peptide solution from the beads. The peptide mixture was dried in a SpeedVac and resuspended in 80 µl extraction buffer (0.1% trifluoroacetic acid, 2% acetonitrile) by sonication. Subsequently, separation of the obtained peptide mixture and mass measurements of peptides and their fragments were performed using an LC–MS system consisting of Evosep One (Evosep Biosystems, Odense, Denmark) coupled to an Orbitrap Exploris 480 mass spectrometer (Thermo Fisher Scientific, Waltham, MA, USA). The obtained results were compared with the NCBI database limited to *J. lividum* using the MASCOT programme (http://www.matrixscience.com/).

The obtained proteomics data have been deposited to the ProteomeXchange Consortium via the PRIDE partner repository and are available via ProteomeXchange with dataset identifier PXD050374 and DOI 10.6019/PXD050374. The list of identified proteins with the number of detected peptides was exported to the Excell MS software (1954 proteins; Supplemental Table S2).

Only protein compositions that overlapped between three independent samples were included in further analysis (932 proteins; Supplemental Table S3). Subsequently, proteins with fewer than 2 mapped peptides were removed from the dataset (resulting in 731 proteins) and the remaining proteins were mapped to the relevant gene ontology (GO) terms using eggNOG-mapper (resulting in 274 proteins) (Cantalapiedra et al. 2021). Finally, these 274 proteins were further analysed for their function in cellular processes using ShinyGO 0.80 limited to the STRING database (Ge et al. 2020), which only found metabolic function assignments for 208 proteins.

### Human skin cell lines and culture conditions

In vitro studies were performed on keratinocytes: HaCaT cells (300493, Cytion, Eppelheim, Germany) cultured in Dulbecco’s modified Eagle’s medium (DMEM) supplemented with 4.5 g/l glucose and 2 mM l-glutamine (VWR, Radnor, PA, USA) and three melanoma cell lines: WM35 (CRL-2807, ATCC, Manassas, VA, USA), WM115 (CRL-1675, ATCC, Manassas, VA, USA) and A375-P (CRL-3224, ATCC, Manassas, VA, USA) cultured in Roswell Park Memorial Institute medium 1640 (RPMI-1640; VWR, Radnor, PA, USA). All culture media were supplemented with 10% (v/v) fetal bovine serum (FBS, Life Technologies part of Thermo Fisher Scientific, Waltham, MA, USA) and antibiotics (100 U/ml penicillin, 0.25 µg/ml streptomycin, Life Technologies part of Thermo Fisher Scientific, Waltham, MA, USA). Cultures were grown at 37 °C in a 5% CO_2_ atmosphere. Cell cultures at 80–90% confluence were treated with trypsin–EDTA solution (0.25%, HaCaT or 0.05%, melanoma; Life Technologies part of Thermo Fisher Scientific, Waltham, MA, USA), diluted to the appropriate density and plated on the surface of the culture vessel.

### Cell metabolic activity assay

The 3-(4,5-dimethylthiazol-2-yl)−2,5-diphenyl tetrazolium bromide (MTT; MilliporeSigma, Burlington, VT, USA) assay was used to assess cell viability according to the following protocol. Cells were seeded on a 96-well plate at a concentration of 2.0 × 10^4^ cells per well in complete DMEM or RPMI-1640 medium and incubated for 24 h at 37 °C and 5% CO_2_. Violacein was then added to fresh culture medium at concentrations ranging from 0.5 to 4.0 µM as an extract or incorporated into EVs, after which the cells were cultured for an additional 24 h under the same conditions. MTT salt was dissolved in PBS (5 mg/ml) and diluted with pure DMEM or RPMI-1640 medium (depending on the cell line) to a final concentration of 0.5 mg/ml. After treatment, the medium containing violacein was removed and replaced with 100 µl of MTT working solution per well, followed by incubation of the plates for 1 h at 37 °C, 5% CO_2_. At the end of the procedure, the medium was discarded, and to dissolve the purple formazan product, 100 µl of DMSO (MilliporeSigma, Burlington, VT, USA) was added to each well and shaken for 15 min at room temperature. The absorbance of the resulting solutions was determined at a wavelength of 570 nm on a microplate reader (Synergy H4, BioTek Instruments, Inc. part of Agilent Technologies, Santa Clara, CA, USA). The results were averaged from four independent experiments and expressed as the relative metabolic activity of treated versus untreated cells. In the 168-h long-term test variant, cultures were performed in 6-well plates with proportional rescaling of both the number of seeded cells and the proportions of other reagents. The value of the half maximal inhibitory concentration (IC_50_) was calculated using the Calculator AAT Bioquest, Inc. (https://www.aatbio.com/tools/ic50-calculator; accessed 28 December 2023).

### Imaging of the cellular internalisation of Nile Red-stained EVs

Nile Red staining of EVs was performed according to a previously published methodology (Mierzejewska et al. 2023). Briefly, 100 µl of EVs were mixed with 4 μl of Nile Red (2 mg/ml in acetone, Carl Roth GmbH + Co. KG, Karlsruhe, Germany) and incubated for 30 min at room temperature in the dark. The samples were then washed 6 times with 500 μl PBS using centrifugal filter units with a 50 kDa cut-off (Amicon Ultra-4 Centrifugal Filter Unit, Merck, Darmstadt, Germany) in spin cycles at 7500 × *g*, 5 min. After centrifugation, the final volume of stained EVs was measured and adjusted to the initial volume with PBS buffer. Cells were seeded at a concentration of 3.2 × 10^4^ cells per sterile round glass coverslip in a 24-well plate and cultured in 400 µl of DMEM or RPMI-1640 medium under standard conditions for 24 h. The medium was then discarded, the cells were gently washed with PBS and 400 µl of Nile Red-stained EVs suspension in DMEM or RPMI-1640 medium (corresponding to 20 µM violacein) was added. Non-treated cells were used as a negative control. The cells were cultured under standard conditions for another 1 h. The cells were then washed with PBS, fixed with 3.7% (w/v) paraformaldehyde (15 min, RT, dark) and washed again with PBS. The nuclei were stained with Hoechst 33342 (Thermo Fisher Scientific, Waltham, MA, USA) diluted in PBS (final concentration of 0.5 μg/ml, 10 min, RT, in darkness). The dye was removed, and the cells were washed twice with PBS. The coverslips were removed from the well and placed upside down on a 5 µl drop of VECTASHIELD Antifade Mounting Medium (Vector Laboratories, Newark, DE, USA) on a microscope slide. Observations were made using a fluorescence microscope (Eclipse Ni, Nikon, Tokyo, Japan) in white, blue (filter block FF01-392/23 nm excitation, FF02-447/60 nm emission) and red (filter block FF01-554/23 nm excitation, FF02-609/54 nm emission) light equipped with a × 60 objective (Plan Fluor objective lens 60 × / 0.85 ∞/0.11 − 0.3 WD 0.40 − 0.31 B, Nikon, Tokyo, Japan).

### Actin cytoskeleton staining

To assess changes in the structure of the actin cytoskeleton, staining was performed according to a previously applied methodology with minor modifications (Sobiepanek et al. 2016). Cells were seeded at a concentration of 3.2 × 10^4^ cells per sterile round glass coverslip in a 24-well plate and cultured in 400 µl of complete DMEM or RPMI-1640 medium under standard conditions for 24 h. The cells were then washed with PBS, 400 µl of EVs-integrated violacein (1.0, 2.0 or 4.0 µM) was added to a fresh culture medium, and the cells were cultured for an additional 24 h under standard conditions. After treatment, the medium was removed and the cells were washed twice with PBS buffer and fixed in 3.7% (w/v) paraformaldehyde with 0.5% (v/v) glutaraldehyde in PBS (MilliporeSigma, Burlington, VT, USA). After 20 min of incubation at 4 °C, the cells were washed with PBS for 5 min and then permeabilized by incubation with 0.2% Triton X-100 in PBS (MilliporeSigma, Burlington, VT, USA) for 15 min at RT. After washing in PBS (5 min, RT) to prevent nonspecific phalloidin binding, the cells were incubated for 30 min at room temperature in a solution containing 0.05% (v/v) Triton X-100 and 1% (w/v) bovine serum albumin (BSA, MilliporeSigma, Burlington, VT, USA). After washing in PBS (5 min, RT), actin filaments were stained with phalloidin diluted in 1% (w/v) BSA with 0.05% Triton X-100 (2 drops/1 ml, ActinRed 555, ReadyProbes Reagent, Thermo Fisher Scientific, Waltham, MA, USA) for 30 min at RT and washed in 0.05% Triton X-100 in PBS (10 min, RT). Chromatin was stained with a Hoechst 33342 dye (0.5 μg/ml in PBS) for 10 min at RT, followed by washing with PBS (10 min, RT). The wet coverslips were placed on a microscope slide with a 5 µl drop of VECTASHIELD Antifade Mounting Medium (Vector Laboratories, Newark, DE, USA), sealed with nail varnish and imaged using a fluorescence microscope (Eclipse Ni, Nikon, Tokyo, Japan) in white, blue (filter block FF01-392/23 nm excitation, FF02-447/60 nm emission) and red (filter block FF01-554/23 nm excitation, FF02-609/54 nm emission) light equipped with a × 60 objective (Plan Fluor objective lens 60 × / 0.85 ∞/0.11 − 0.3 WD 0.40 − 0.31 B, Nikon, Tokyo, Japan).

### Apoptosis and necrosis assay

The RealTime-Glow Annexin V Apoptosis and Necrosis Assay (Promega, Madison, WI, USA) was used to verify the mechanism of action of violacein encapsulated in EVs, following the manufacturer's recommendations listed below. Cells were seeded and treated with EVs-Vio in the same manner as for the viability assay. During cell treatment, 50 μl of the appropriate dilution of violacein and 50 μl of the previously prepared detection reagent were added to each culture well. The detection reagent was prepared by adding to the medium in the following order NanoBit® Substrate, CaCl_2_, Necrosis Detection Reagent, Annexin V-SmBiT and Annexin V-LgBiT, with a final × 500 dilution of each reagent. The plate was vortexed (30 s, 500 rpm, in darkness) and then incubated under standard conditions. Luminescence and fluorescence (485/525 nm) were performed at the 3rd, 6th, 9th and 24th hours of the experiment using a Synergy H4 microplate reader (BioTek Instruments, Inc. part of Agilent Technologies, Santa Clara, CA, USA).

### Statistical analysis

The data were presented as the means ± standard deviations (SD) from at least three independent experiments performed in triplicate. The statistically significant differences were analysed by a one-way analysis of variance (ANOVA) using OriginPro 8 (OriginLab, Northampton, MA, USA). A probability value (*p*-value) < 0.05 was considered statistically significant.

## Results

### Characterisation of EVs produced by *J. lividum*

**Fig. 1:**
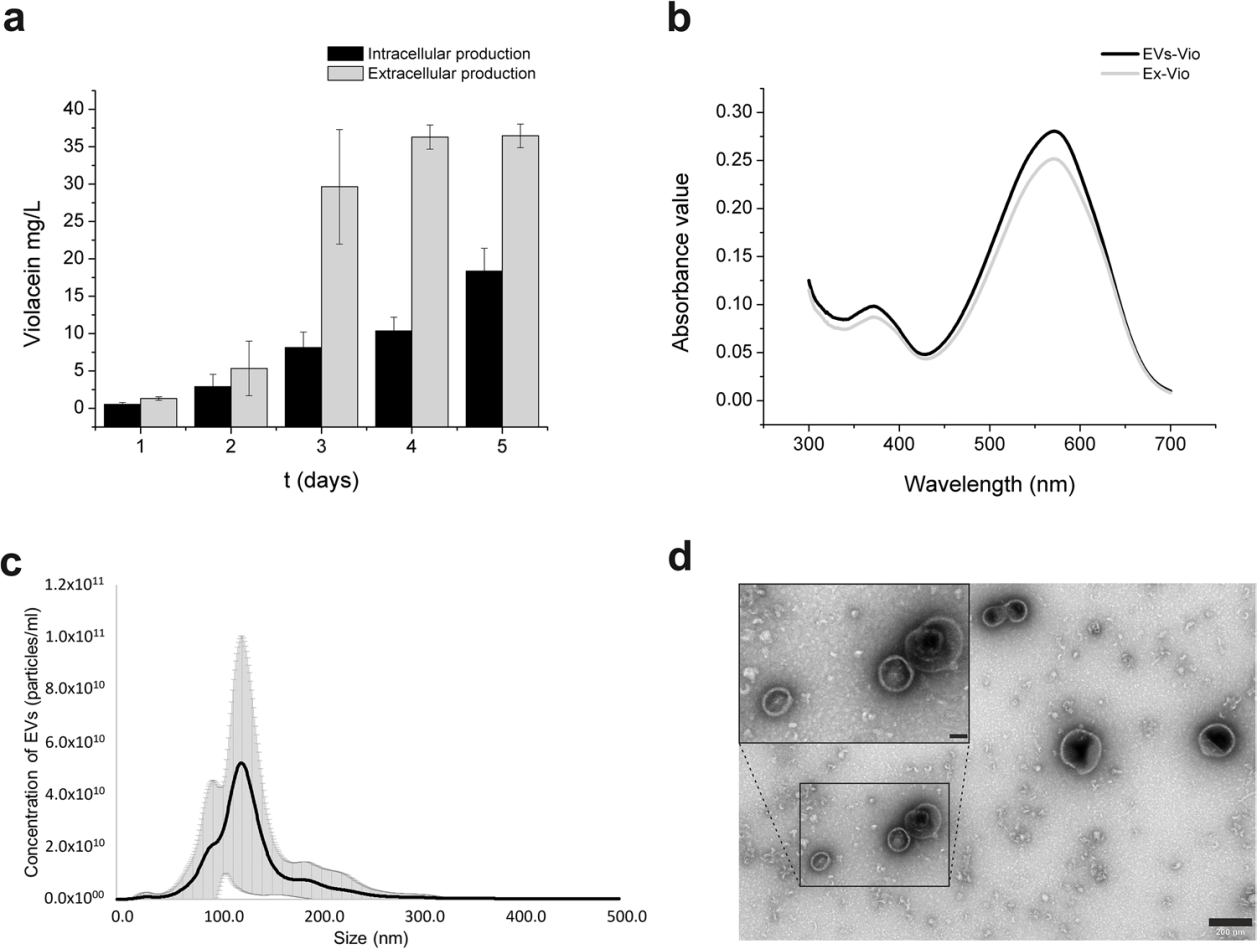
Characteristics of EVs produced by *J. lividum.* **a** Bioproduction efficiency of violacein enclosed in EVs vs. accumulated in the cell biomass. **b** Representative spectra of the crude methanol extract of violacein and violacein from EVs. **c** Concentration and size distribution of EVs determined by NTA. **d** TEM image of *J. lividum* EVs with scale bars of 200 nm and 50 nm, respectively

The bacterial strain used in this work can produce violacein both inside the cell and in the form of characteristic extracellular vesicles. Obtaining violacein from each of these forms is different, namely, violacein can be isolated from the cell sediment using solvents, whereas the separation of EVs from the post-culture medium is based on the use of sequential ultrafiltration (Supplemental Fig. S1). Starting with the characterisation of EVs from *J. lividum*, the contribution of these structures to the translocation of violacein produced by the cells was investigated. This involved testing which of the two bioproduction routes for violacein was more effective. To monitor the production efficiency of violacein in the intracellular form and encapsulated in EVs, samples were taken daily from the production culture. Analysis of the results showed that the extracellular production of violacein encapsulated in the form of EVs was more efficient (Fig. 1a). This difference was particularly evident after the third day of cultivation (8.13 ± 2.07 mg/l vs. 29.64 ± 7.66 mg/l). A wavelength scan from 300 to 700 nm was performed to compare violacein extracted from the cells with methanol and exported in EVs. As shown in Fig. 1b, the maximum absorption wavelength of both samples was 572 nm, which is consistent with the maximum absorption wavelength previously reported for violacein (Ahmad et al. 2012). This maximum absorption peak is a characteristic feature of the violacein pigment, suggesting that the dye in both samples has similar properties.

The size and concentration of EVs in the samples were determined using nanoparticle tracking analysis (NTA). Purified EVs were obtained at an average of approximately (2.83 ± 0.12) × 10^12^ particles/ml with an average size of 124.07 ± 3.74 nm and a range between 48.5 and 268.5 nm (Fig. 1c).

Finally, to obtain an overview of the morphology of the EVs samples, extracellular vesicles were visualised using transmission electron microscopy. TEM images confirmed the occurrence of large and small vesicles (Fig. 1d).

### Identification of proteins isolated from *J. lividum *EVs

The composition and charge of bacterial vesicles are highly variable, which significantly influence the physiological functions performed by a particular type of EVs. One of the basic components of EVs, in addition to lipids, are proteins, and it was decided to focus first on understanding their content. Because the protein precipitate isolated from the EVs was always contaminated with coloured violacein, it was not possible to determine the amount of protein in the precipitate using the most known methods based on spectrophotometry. Therefore, the samples were compared volumetrically, and efforts were made to purify EVs from the same amount of culture each time so that comparable volumes of purified EVs were obtained in the end.

**Fig. 2:**
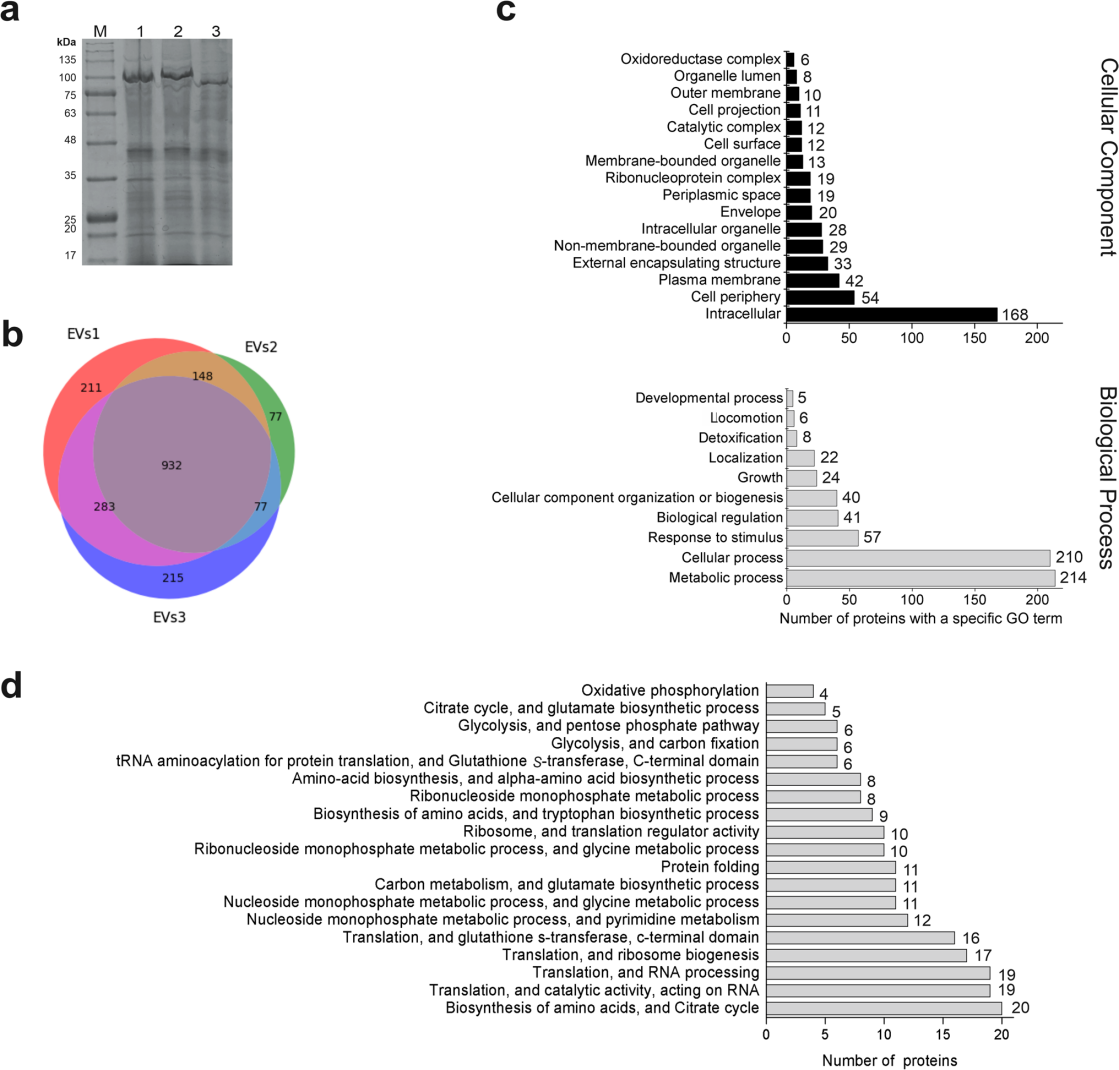
Characterisation of the proteome of EVs produced by *J. lividum*. **a** SDS-PAGE analysis of protein precipitates from three EV isolates. Track M is the ladder mass, whilst tracks 1, 2 and 3 are protein samples from three independent isolations. **b** Comparison of the protein composition profiles of the three EVs isolates. **c** The most common predicted cellular localisation and involvement in biological processes of proteins associated with EVs based on gene ontology analysis. Rare variants (those with fewer than 5 assignments) are not shown. **d** Pathways involving proteins common to the three EVs isolations. The identified proteins were submitted to the ShinyGO 0.80 analysis tool, restricted to the STRING database and *J. lividum* species. Shown are top 19 pathways in which EVs proteins are most likely to be involved

Proteins isolated from the same volume of vesicles from three independent experiments were analysed by SDS-PAGE. The observed pattern of bands from the three isolates was very similar: only in the case of proteins migrating at approximately 100 kDa, there was a noticeable difference in the band intensity for isolate number 3 (Fig. 2a). The protein precipitates from the EVs were further analysed by LC–MS/MS to determine their protein content. We identified a total of 1954 proteins (a complete list can be found in Supplemental Table S2). The protein composition that overlapped between three samples was visualised using a Venn diagram (Fig. 2b), and only the common proteins (932), were included in further analysis (Supplemental Table S3). Subsequently, proteins with fewer than 2 mapped peptides were removed from the dataset (resulting in 731 proteins) and the remaining proteins were mapped to the relevant GO (gene ontology) terms. The resulting 274 proteins were further analysed for their function in cellular processes using ShinyGO 0.80 (Ge et al. 2020), which found metabolic function assignments for only 208 proteins. It is noteworthy that the 100 kDa cut-off filters we used may leave some contaminants such as proteins above 100 kDa (assuming that smaller proteins remain in the sample only when bound to EVs). We have therefore carefully considered the size of the proteins that were mapped to the relevant gene ontology terms. The analysis showed that only about 10% of these proteins had a mass greater than 100 kDa i.e. could be a contaminant resulting from the applied purification process. Looking at the assigned GO terms, the highest number of matches was found for proteins annotated as intracellular (168). However, a more careful analysis and summation of all proteins assigned to membranes and external cellular structures revealed that they were even more numerous (189) (Fig. 2c). The *J. lividum* EVs proteins were mainly associated with metabolic or cellular processes (Fig. 2c). Bioinformatic analysis revealed that in addition to pathways related to oxidative phosphorylation, the other detected pathways were related to basic intracellular processes that are common to all living organisms, such as amino acid biosynthesis, the citrate cycle and translation. In addition, the identified proteins were involved in nucleoside and ribonucleoside monophosphate metabolism, carbon metabolism, glycolysis and protein folding (Fig. 2d).

### Interaction of EVs produced by *J. lividum* with human skin cells

**Fig. 3:**
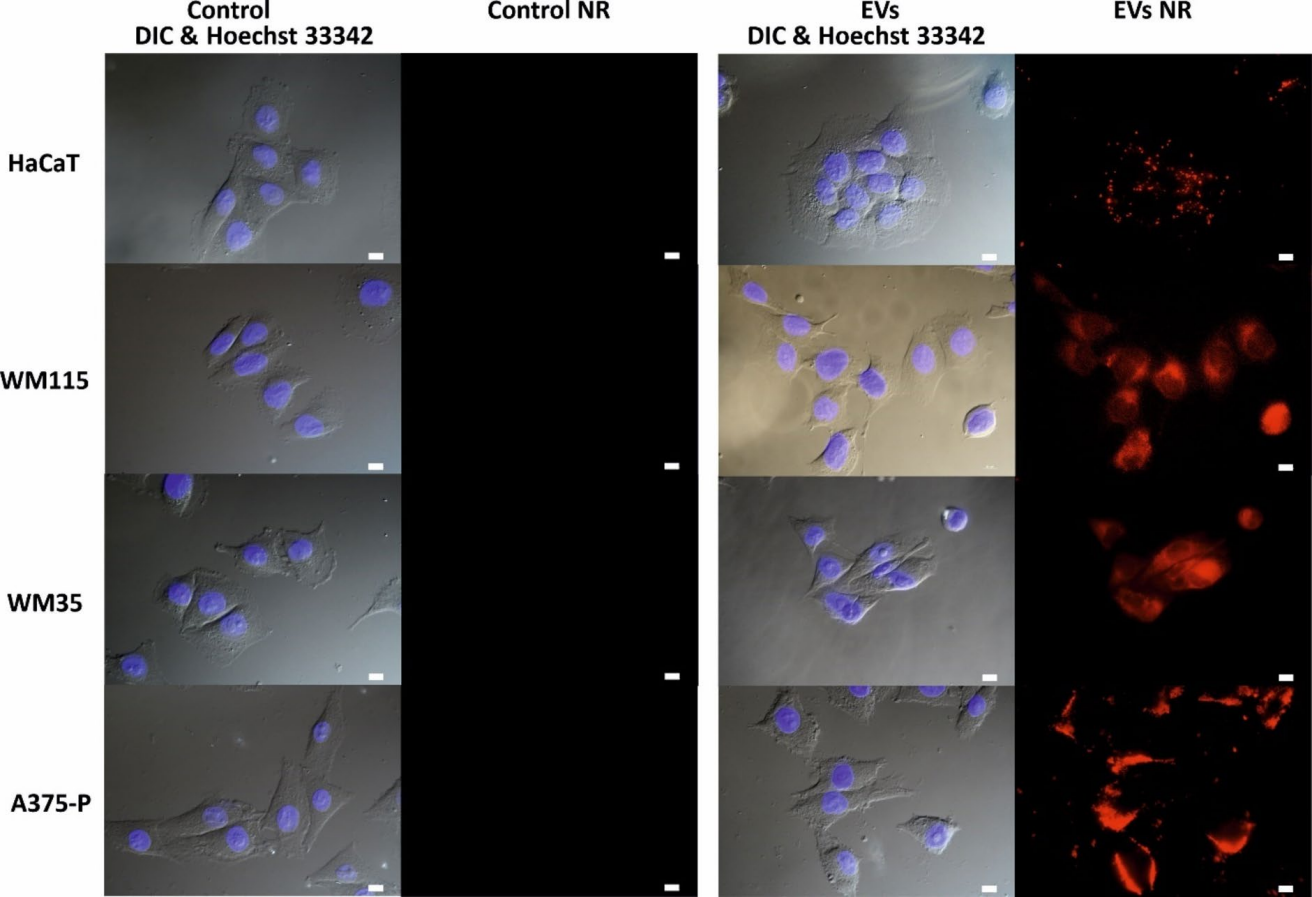
Interaction of stained *J. lividum* EVs with human skin cells of HaCaT keratinocytes and melanoma: WM115, WM35 and A375-P lines imaged by fluorescence microscopy. EVs NR—vesicles stained with a red-fluorescent Nile Red dye; untreated cells were used as a control; cell nuclei were stained with Hoechst 33342; scale bar, 10 µm

Research in recent years has shown that EVs are versatile intercellular and interspecies transporters of bioactive molecules. Therefore, the next goal was to elucidate whether bacterial EVs-containing violacein could interact with human skin cells. This study was conducted using human keratinocyte HaCaT cells, which are normal skin cells, and cancer cells from different stages of melanoma progression (WM35, WM115 and A375-P). EV particles were stained with the lipophilic dye Nile Red so that at the end of the staining process, the dye that contacted the mammalian cells was present only in the vesicles. Skin cells were incubated with stained EVs for 1 h and then imaged under a fluorescence microscope. Subsequently, cell nuclei were stained with Hoechst 33342 for cell localisation. As a result, we observed a marked change in the red fluorescence of the cells due to the lipophilic dye. This visually indicated that the *J. lividum* EVs were in close contact with human skin cells and were internalised by these cells (Fig. 3).

### Cellular metabolic activity in response to different forms of violacein application

**Fig. 4:**
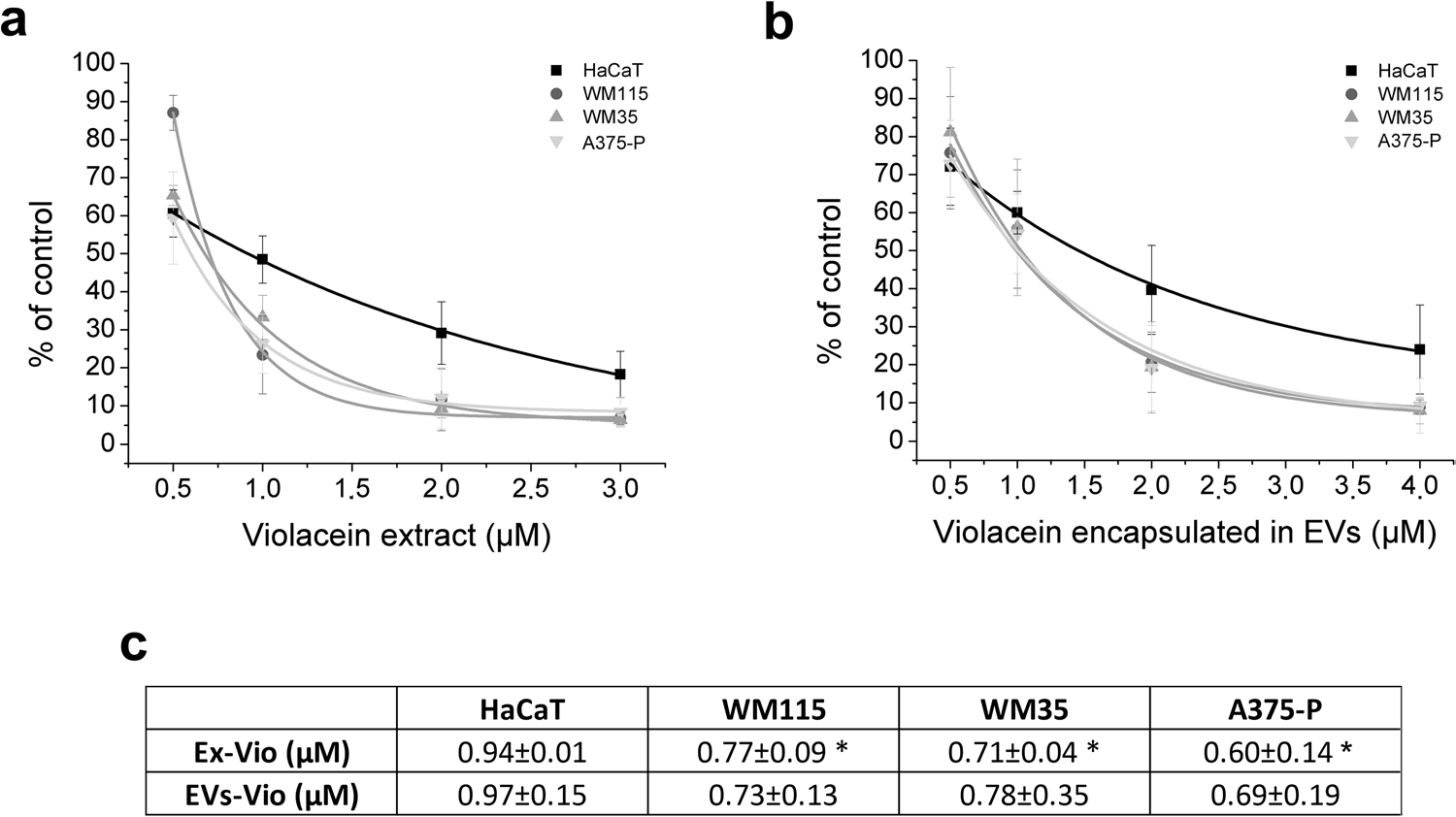
Changes in the metabolic activity of cells in response to different forms of violacein application (MTT assay). **a** The effect of the methanol extract of violacein (Ex-Vio) on skin cells. **b** The effect of violacein associated with EVs (EVs-Vio) on skin cells. **c** IC_50_ values calculated for both forms of violacein. All symbols reflecting the activity of a given concentration of violacein were connected by a line obtained by polynomial regression. Each tested variant was compared with the untreated control cells and expressed as a percentage of the control; mean ± SD values were averaged from 3 independent biological experiments. *Statistical significance (*p*-value < 0.05) of the melanoma cell line response versus the HaCaT cell line response

Having confirmed that extracellular vesicles secreted by *J. lividum* can interact with skin cells, the next task was to test whether bacterial EVs were capable of transferring their cargo (violacein) to human skin cells. To this end, the metabolic effect of violacein administered in the encapsulated form of EVs was compared with the effect of the methanolic extract of this compound (Fig. 4). For this goal, a standard MTT test was used to check the activity of mitochondrial dehydrogenase. The cells were exposed to violacein (0.5–4 µM) for 24 h prior to the test. The change in the metabolic activity of the cells was expressed in relation to that of the untreated cells, after confirming that the solvent itself had no effect. The IC_50_ values were calculated based on the obtained results.

**Fig. 5:**
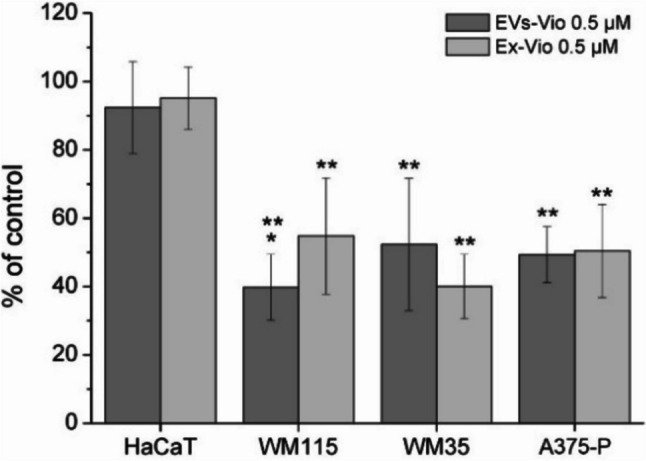
Metabolic activity of cells in response to the application of different forms of violacein after long-term incubation (MTT assay). A violacein concentration of 0.5 µM and a 7-day incubation were used. Each tested variant was compared with the untreated control cells and expressed as a percentage of the control; the mean ± SD values are from 3 independent experiments. *Statistical significance (*p*-value < 0.05) of EVs-Vio vs. Ex-Vio; **statistical significance (*p*-value < 0.05) of melanoma cell line response vs. HaCaT cell line response

The IC_50_ values were in the range of µM concentrations from 0.60 to 0.94 µM in the case of the violacein extract, and from 0.69 to 0.97 µM in the case of the EVs-containing violacein (Fig. 4c). Statistical analysis of the results showed that there were no significant differences between the effects of the extract and violacein encapsulated in EVs. This means that the effect of violacein is comparable regardless of the form of its application. However, it is worth noting that the shape of the response curve (obtained after applying polynomial regression) and the IC_50_ values for HaCaT cells differ from the curves and the IC_50_ values obtained for tumour cells, which may indicate some dissimilarity in the mechanism of this compound action (Fig. 4a, b). The difference between melanoma and keratinocyte cells was particularly evident after the use of a low dose of the compound for a prolonged period of 7 days. In this experiment. it was not possible to use concentrations equal to or exceeding the IC_50_ values due to rapid cell death; therefore, it was decided to use lower concentration i.e. 0.5 μM. Taking into account the EN ISO 10993–5:2009 standard according to which a cytotoxic effect is only considered when cell viability is reduced by more than 30%, in our experimental set-up, keratinocytes which are the dominant component of the epidermis, proved to be resistant to the effects of violacein, whilst melanoma cells were eliminated. Again, no significant differences were observed between the two forms of violacein in the case of HaCaT, WM35 and A375-P lines (Fig. 5). Line WM115 was statistically more suppressed by EVs-Vio (Fig. 5).

### Characteristics of violacein activity in the form of *J. lividum* vesicles

**Fig. 6:**
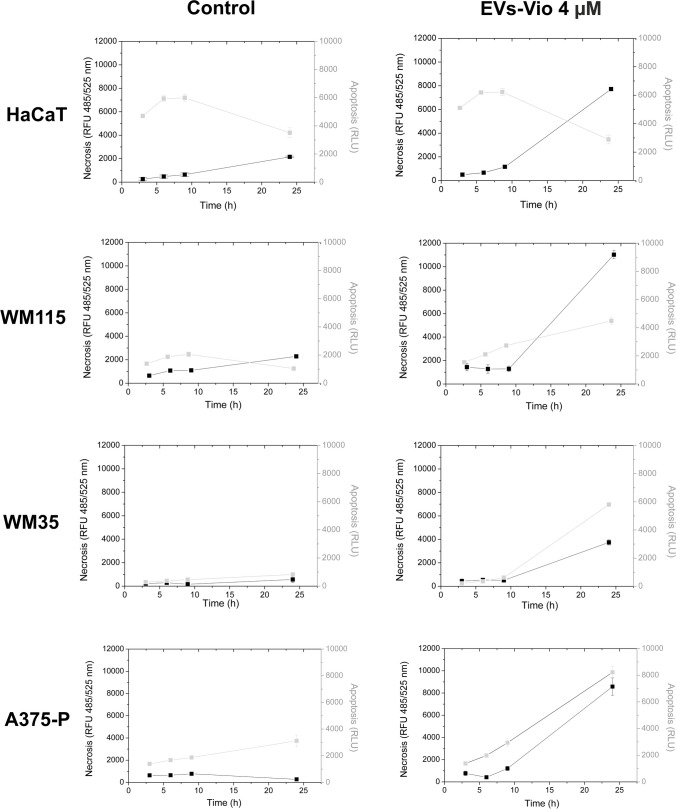
Identification of apoptosis and necrosis processes in HaCaT and melanoma cells after treatment with 4 µM violacein in the form of EVs. The test compared the luminescence signal (associated with apoptosis) and fluorescence signal (associated with necrosis). Luminescence in relative light units (RLU) and fluorescence in relative fluorescence units (RFU) are plotted against the time of measurement. The assay was performed in three independent experiments

The results of the experiments described above confirmed that the metabolic activity of human skin cells under the influence of violacein, administered in either form, undergoes comparable changes. In addition, in melanoma cells, apoptosis has already been identified as the mechanism of cell death that occurs under the action of the extract of violacein (Gonçalves et al. 2016). Therefore, in the next steps (analysis of cell death mechanisms and F-actin organisation), we focused on studying the compound encapsulated in the form of EVs. Our plan was to compare the results with widely available data in the literature for the extract of the dye. The RealTime-Glow Annexin V Apoptosis and Necrosis Assay was used to confirm the mechanism of cell death. During the experiment, changes in the culture following the addition of EVs were recorded at fixed time points. The concentrations of violacein encapsulated in EVs used in the experiment corresponded to the concentrations of violacein used in the MTT assay, but in order to ensure a visible dye effect, their range was above the measured IC_50_ values i.e. 1, 2 and 4 µM (Supplemental Fig. S4). The most pronounced changes were observed at the highest (4 µM) concentration of the compound; therefore, these results are included in this paper (Fig. 6). The test compares the luminescence (associated with apoptosis) and fluorescence (associated with necrosis) signals. In living cells, the cell membrane is asymmetric, and one of its components, phosphatidylserine, is found almost exclusively on its inner side. When apoptosis occurs in the cell, phosphatidylserine moves to the outer part of the membrane, where it binds to annexin V, which is linked to the corresponding luciferase subunit, causing it to glow. As a result of necrosis, the membrane is disrupted, and a fluorescent reagent binds to the DNA. Apoptosis occurs when an increase in fluorescence (necrosis) follows an increase in luminescence (apoptosis) (Kupcho et al. 2019). The obtained curves show that apoptosis occurs in melanoma lines, but in HaCaT cells, the mechanism of death is different. In keratinocytes, the luminescence signal is constant regardless of the application of the compound; only the fluorescence signal, indicating necrosis, increases over time. In melanoma cells, both apoptotic and necrotic signals increase after violacein application (Fig. 6). In order to better document the apoptosis observed in cell culture after treatment with EVs-Vio, we have provided in the supplementary materials a series of photographs showing the morphological changes that indicate that programmed cell death has occurred (Supplemental Fig. S5).

**Fig. 7:**
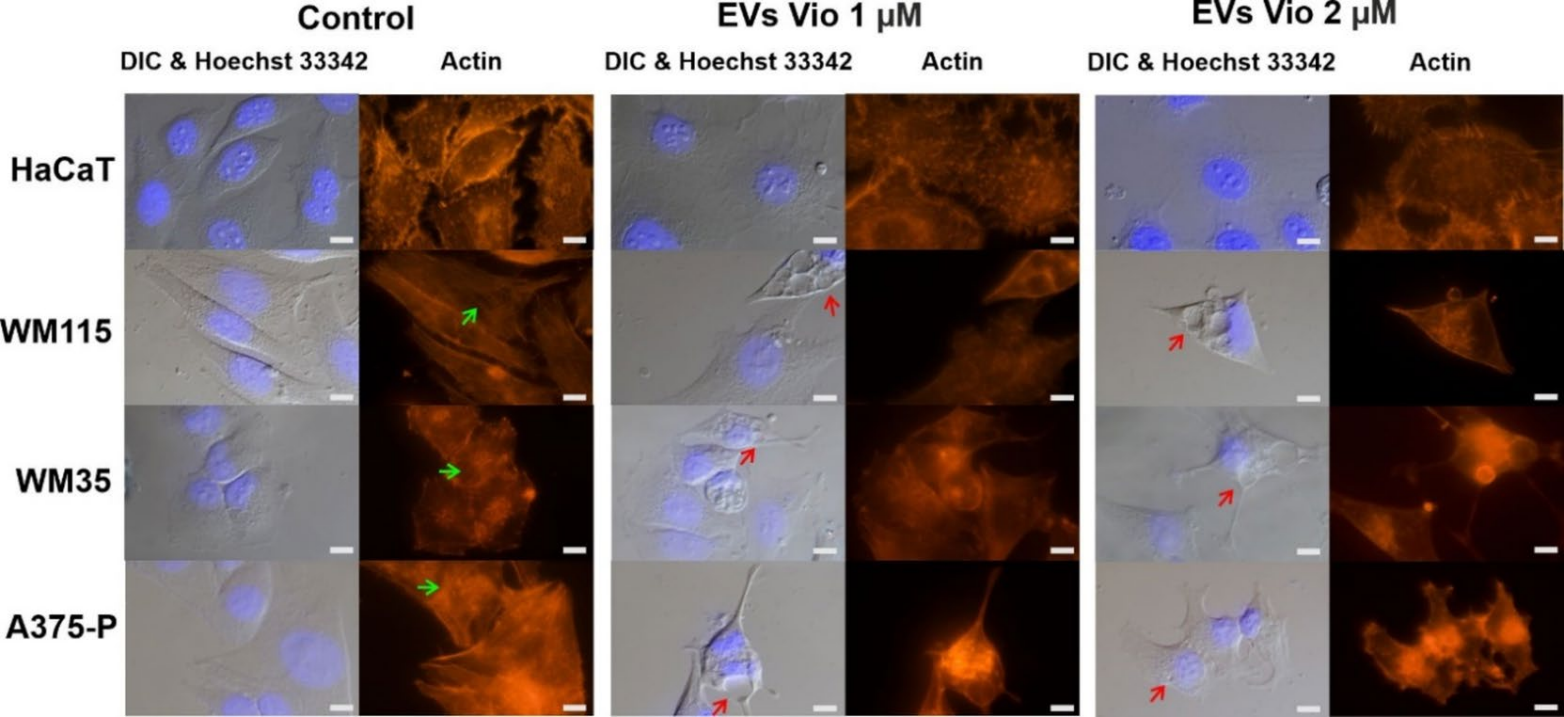
Fluorescence microscopy analysis of F-actin organisation in HaCaT and melanoma cells after treatment with two concentrations of violacein delivered in the form of EVs (EVs-Vio). Untreated cells served as control. Actin filaments were stained with phalloidin (ActinRed 555); cell nuclei were stained with Hoechst 33342; scale bar, 10 µm; red arrows indicate vacuolization; green arrows indicate long actin filaments that cross-linked whole cells

Finally, we investigated the morphological changes in the cells treated with violacein in the form of EVs. As before, to ensure a visible dye effect, their range was above the measured IC_50_ values i.e. 1, 2 and 4 µM. Unfortunately, after 4 µM violacein treatment, the staining procedure (which includes a number of rinse cycles) could not be carried out properly because the binding of the cells to the surface was too weak, more than 80% of the cells were dead and not attached to the surface. The morphological changes of cells imaged by light microscopy with differential interference contrast (DIC) were examined, and the actin cytoskeleton stained with phalloidin bound to the appropriate fluorophore was imaged (Fig. 7). Phalloidin staining of untreated cells revealed a dense actin mesh network mainly in the central cytoplasm region (keratinocytes) and a network of long actin filaments that cross-linked whole cells, resembling actin stress fibres (melanoma). Despite the administration of a 1 or 2 µM dose of violacein to keratinocytes, there were no significant changes in the structure of the actin cytoskeleton. In contrast, melanoma cells showed vacuolization, changes in cell shape and depolymerisation of long actin filaments, which were no longer visible (Fig. 7).

## Discussion

The human body is made up of approximately 70% water; therefore, drug molecules must be in a dissolved form to achieve adequate bioavailability. In addition, aqueous drug solutions are preferred for pharmacological, toxicological and pharmacokinetic studies at both the preclinical and clinical stages. Thus, poor aqueous solubility not only limits the biological application of a drug but also poses a challenge to its pharmaceutical development. As a result, the search for methods to increase solubility has been a constant feature of pharmaceutical research for several decades (Kalepu and Nekkanti 2015; Kumari et al. 2023). Violacein is a substance with great potential, but its lack of solubility in water severely limits its capabilities for widespread practical use. As it has been already demonstrated, violacein remains stable in an aqueous environment when is enclosed in the *C. violaceum* EVs (Choi et al. 2020; Batista et al. 2020). The present study provides a detailed characterisation of violacein-containing EVs secreted by another dye producer, *J. lividum*, and their exemplary use in the treatment of melanoma cells. Using a range of research techniques including NTA, TEM and LC–MS/MS, the size, morphology and protein composition of *J. lividum* EV probes were determined. Similar to *C. violaceum*, *J. lividum* PCM 3520 has the ability to produce violacein in a form that is trapped inside the cell and released into the culture medium inside EVs. However, in the case of *C. violaceum*, intracellular production accounts for almost 92% of the total violacein content in the culture (Choi et al. 2020), whereas *J. lividum* exports violacein into the medium with a much greater efficiency, averaging 71.7% (Fig. 1a). In terms of size, *J. lividum* EVs fall within the range described for *C. violaceum* vesicles (Choi et al. 2020).

In the NCBI database, the reference genome for *J. lividum* is the genome assembly ASM1337204v1: strain EIF1 with a size of 6.4 Mbp. In terms of the 16S rRNA coding sequence, this species was mostly similar to the PCM 3520 strain (Supplemental Table S1). The *J. lividum* genome contains 5551 coding sequences of which 122 are rRNAs, 93 tRNAs and 1 tm-RNA (Friedrich et al. 2020). In this light, the 1954 proteins identified by LC‒MS/MS in the EVs of *J. lividum* PCM 3520 would represent approximately 36.6% of the proteins encoded in the genome of the reference sequence for this bacterial species. The specific details of the number of genome-encoded proteins that end up in bacterial extracellular vesicles are an individual property of a given strain, variable within species (Zwarycz et al. 2020) and dependent on a number of factors, including the mechanism of biogenesis (Zavan et al. 2023).

The accuracy of the pathway analysis depends primarily on the quality of the annotations found in the existing databases, such as the correct annotation of proteins, the proportion of a set of proteins in the pathways, the topology of the pathways, and the presence of proteins in the global network. However, these data are far from complete, especially for relatively poorly studied organisms such as *J. lividum*. Amongst the currently available tools for pathway analysis, only the Search Tool for the Retrieval of Interacting Genes/Proteins (STRING) database (Szklarczyk et al. 2023) was able to find a reference to *J. lividum.* However, even in this case, only 208 proteins could be analysed. Therefore, the results presented in this study (available via ProteomeXchange) will need to be further refined as existing databases are developed. However, what is striking in this analysis is the high proportion of proteins with intracellular localisation that are involved in many basic cellular processes such as amino acid biosynthesis, the citrate cycle or translation. This situation, together with the identification of plasma membrane proteins (Fig. 2c, d), led to the hypothesis that the extracellular vesicles secreted by *J. lividum* PCM 3520 are of the outer-inner membrane vesicle (O-IMV) type, whose biogenesis originates from cell lysis induced by phages or environmental stress (Nagakubo et al. 2020; Fang et al. 2022; Zavan et al. 2023). The cell lysis model proposes that O-IMVs are generated as a result of stress, which can be induced by antibiotic treatment or exposure to detergents and can lead to a breakdown in membrane integrity and a subsequent release of large amounts of cytoplasmic and periplasmic contents, including membrane fragments (Devos et al. 2017; Charpentier et al. 2023). In the case of *J. lividum* EVs, this type of biogenesis is further supported by the fact that the secretion of *J. lividum* EVs coincides with the production of violacein inside bacterial cells (Fig. 1a), and violacein is known to have a strong disruptive effect on cell membranes (de Souza et al. 2017; Cauz et al. 2019; Gupta et al. 2021). In addition, violacein has already been suggested as a factor stimulating outer membrane vesicles (OMVs) release in *C. violaceum* and *Eschericha coli* (Batista et al. 2020).

A number of pathways in which EV-loaded *J. lividum* proteins are involved have been identified by bioinformatic analysis (Fig. 2d). These pathways were mainly related to basic intracellular processes such as amino acid biosynthesis, the citrate cycle, translation, nucleoside and ribonucleoside monophosphate metabolism or carbon metabolism, and glycolysis. Interestingly, many of the identified pathways have counterparts in an analysis performed on data from 29 Gram-negative bacterial species, in which a functional classification of the Clusters of Orthologous Groups (COGs) represented in the EV proteomes revealed that highly overrepresented COGs categories are associated with amino acid metabolism and transport, energy production and conversion, translation, nucleotide metabolism and transport or carbohydrate metabolism and transport (Stathatos and Koumandou 2023).

Using skin cell lines, we confirmed that *J. lividum* EVs can react with mammalian cells and transfer violacein into the cells (Fig. 3). This was not very surprising given that the OMVs of many Gram-negative bacteria are recognised as a generalised secretion pathway, transferring their cargo to other bacteria as well as to eukaryotic cells (Thapa et al. 2023; Gan et al. 2023). In addition, the activity of violacein administered in this form was maintained, which is supported by the results of the viability tests, which showed no significant differences between the effects of the extract and violacein encapsulated in EVs (Figs. 4 and 5). The exception was the response of the line WM115 which was statistically more suppressed by EVs-Vio, although the difference was only about 15% (Fig. 5). The lines used in the experiment are from different stages of melanoma progression and also have different molecular characteristics (Supplemental Table S4). The molecular difference of the cell line from the vertical growth phase may be related to its slightly different response to the violacein form. The effect of other components of EVs, such as proteins/other metabolites, cannot be excluded. Therefore, the contribution of the latter factor requires further in-depth research. To date, the use of some artificial delivery devices loaded with violacein has been suggested (Durán et al. 2021). Interestingly, the activity of violacein in the complexes depended on the type of complex and the cell line tested e.g. in the case of β-cyclodextrin delivery system and V79 fibroblasts, the cytotoxicity of violacein was reduced (De Azevedo et al. 2000), whereas the cytotoxicity with respect to leukaemia HL60 cells was increased in the presence of the β-cyclodextrin molecules (Melo et al. 2003).

What particularly caught our attention was that the shape of the response curve for HaCaT cells differed from the curves obtained for tumour cells (Fig. 4a, b). It is worth noting that it has already been shown that cells that have not undergone tumour transformation respond differently to the effects of violacein than do cancer cells (Mojib et al. 2011). This raises the hope of finding conditions of violacein action under which cancer cells would be precisely eliminated from the epidermis. In fact, such differentiation was achieved after the use of a low dose of the compound for a prolonged period of 7 days (Fig. 5). Under these conditions, melanoma cells were selectively eliminated. To the best of our knowledge, this is the first report comparing human skin cell lines in this context. However, in a mouse skin model, in which B16F10 melanoma cells were compared with normal C50 keratinocytes, inhibition of the growth of only cancer cells was observed during a 72-h experiment in which cells were treated with 0.5 µM violacein-like purple pigment (Mojib et al. 2011).

Finally, we used the RealTime-Glow Annexin V Apoptosis and Necrosis Assay and visual examination of cell morphology and actin cytoskeleton changes under violacein treatment to characterise the activity of the compound applied in the form enclosed in *J. lividum* EVs (Figs. 6 and 7). We found that apoptosis occurred only in melanoma cell lines, and in HaCaT cell lines, the mechanism of cell death differed. Furthermore, no significant changes in cell morphology or cytoskeletal structure were observed in HaCaT cells. In contrast, in melanoma cells, we observed vacuolization, changes in the cell shape and depolymerisation of the actin filaments. The mechanism of action of violacein at the molecular level in mammalian cells is still fragmentary and selective, despite the large amount of work on the subject (De Souza et al. 2005; Durán et al. 2016, 2021; Masuelli et al. 2016; de Souza et al. 2017). It appears to be specific to cell type and molecular characteristics (Leal et al. 2015; Tsukimoto et al. 2021; Neroni et al. 2022). For example, amongst the already tested leukaemia cell lines, violacein showed selective cytotoxicity against HL60 and TF-1 cells, but the pathways leading to cell death differed in each case. In HL60 cells, exposure to violacein led to apoptosis. The authors noted phosphorylation of p38 MAP kinase, increased levels of the nuclear factor κB pathway and activation of caspases (Ferreira et al. 2004). It was also found that this effect was associated with specific activation of tumour necrosis factor receptor 1. On the other hand, TF-1 cells did not appear to follow the canonical apoptotic pathway and/or autophagy, since biomarkers of both types of cell death were not significantly affected by violacein (Queiroz et al. 2012). To date, inhibition of autophagy by violacein has been observed in RAS-mutant metastatic melanoma, which in turn leads to apoptosis, what is also consistent with our observations (Gonçalves et al. 2016). Similarly, cytoplasmic vacuolization as a result of violacein treatment has been reported previously (Queiroz et al. 2012; Tsukimoto et al. 2021). Cytoplasmic vacuolization is a well-known morphological phenomenon observed in mammalian cells after exposure to a variety of chemicals and bioactive agents. Vacuolization often accompanies many types of cell death such as apoptosis, autophagy, necrosis, paraptosis and others; however, its role in cell death processes remains unclear (Zhang et al. 2010; Aki et al. 2012; Shubin et al. 2016). In the case of violacein, the vacuolization it induces may be the result of changes that occur in cells after they have entered the programmed cell death pathway, but a detailed explanation of this phenomenon would require further research. A detailed study of the response of the actin cytoskeleton to violacein treatment is unprecedented in the context of mammalian cells. To date, the effect of violacein on actin filament structure has only been reported in the malaria parasite (Wilkinson et al. 2020). Nevertheless, as previously suggested, violacein can inhibit brain tumour cell migration, likely as a consequence of disrupting subcellular domain structures of the actin filament network, including lamellipodia and filopodia, leading to a rounded cellular phenotype that compromised the motility of these cells (Mehta et al. 2015).

Analysing the interaction of violacein in both forms with skin cells raises the question of how to exclude the influence of impurities such as metabolites, proteins or other additives that are potentially present in crude extracts or EVs? HPLC analyses of crude extracts obtained from *J. lividum* PCM 3520 (Supplemental Fig. S3) showed that, in addition to violacein and deoxyviolacein, other compounds sensitive to 575 nm detection were below 0.6%. However, as in the case of *Chromobacterium* sp. (Menezes et al. 2013), it cannot be excluded that some other component may contribute to the ultimate anti-cancer effect of the PCM 3520 extract. A detailed investigation of such a hypothetical component would require detailed research well beyond the scope of this publication. It is much more difficult to rule out other active factors in EVs. However, it is noteworthy that studies on mutant strains of *C. violaceum*, which are able to secrete EVs without the dye component, have shown that violacein is the predominant active agent of bacterial vesicles (Batista et al. 2020). It seems that in the case of the strain PCM 3520, the situation may be analogous, as indicated by the remarkable similarity between the activity of violacein applied in extract form and that of EVs (Fig. 4).

In summary, our report focuses on the investigation of a new carrier for violacein, an active substance with a previously demonstrated broad spectrum of activity, including anticancer and antimicrobial effects. The results obtained allowed us to conclude that it is possible to purify violacein from the strain *J. lividum* encapsulated in EVs, which has antitumour activity comparable to that of the methanol extract of this compound. The obtained EVs were characterised in terms of their size, morphology and protein composition, which represents a milestone for their future potential application. Using a human skin model, we demonstrate that it is possible to choose the concentration of the compound and the time of its action such that normal cells, like keratinocytes, the dominant component of the epidermis, are resistant to the effects of violacein, whilst melanoma cells are eliminated from the culture. In addition, our results confirmed, in light of previously published research, that the characteristics of the activity of violacein applied in the encapsulated form of EVs are similar to those of its organic solvent extract. Taking all of the above into account, the final conclusion of this study is that EVs from *J. lividum* are promising candidates for use as effective and water-soluble carriers of violacein, expanding the dye’s potential use in the treatment of cancer. In addition, their production is based on a simple filtration method, which is much more environmentally friendly than obtaining dye by chemical extraction, which further enhances *J. lividum* EVs application potential.

## Supplementary Information

Below is the link to the electronic supplementary material.

Supplementary file1 (PDF 798 KB)

Supplementary file2 (XLSX 224 KB)

## Data Availability

All the data generated in this study are available in the main text or Online Resources (Supplemental Figs. S1–S5 or Tables S1–S4). Moreover, the MS datasets generated and analysed during the current study are available from ProteomeXchange in the PRIDE repository (https://www.ebi.ac.uk/pride/), with the identifier PXD050374 and DOI 10.6019/PXD050374.
