# Supplementary material for: Extracellular vesicles of Janthinobacterium lividum as violacein carriers in melanoma cell treatment
Source: Appl Microbiol Biotechnol. 2024 Dec 5;108:529. doi: 10.1007/s00253-024-13358-1 (PMC11621134; doi:10.1007/s00253-024-13358-1)
Supplement: Supplementary file 1 [file 253_2024_13358_MOESM1_ESM.pdf]

Applied Microbiology and Biotechnology

Supplementary Material

Extracellular vesicles of *Janthinobacterium lividum* as violacein carriers in melanoma cell treatment

Patrycja Kowalska[1,2], Jolanta Mierzejewska[1], Paulina Skrzeszewska[1], Aleksandra Witkowska[1], Katarzyna Oksejuk[1], Ewa Sitkiewicz[3], Mariusz Krawczyk[4], Magdalena Świadek[5], Agata Głuchowska[5], Klaudia Marlicka[1,2], Anna Sobiepanek[1], Małgorzata Milner-Krawczyk[1*]

[1] Chair of Drug and Cosmetics Biotechnology, Warsaw University of Technology, Warsaw, Poland

[2] Doctoral School Warsaw University of Technology, Warsaw, Poland

[3] Mass Spectrometry Laboratory, Institute of Biochemistry and Biophysics, PAS, Warsaw, Poland

[4] Genomed S.A., Warsaw, Poland

[5] Nencki Institute of Experimental Biology, Polish Academy of Science, Warsaw, Poland

* corresponding author`s email: malgorzata.krawczyk@pw.edu.pl

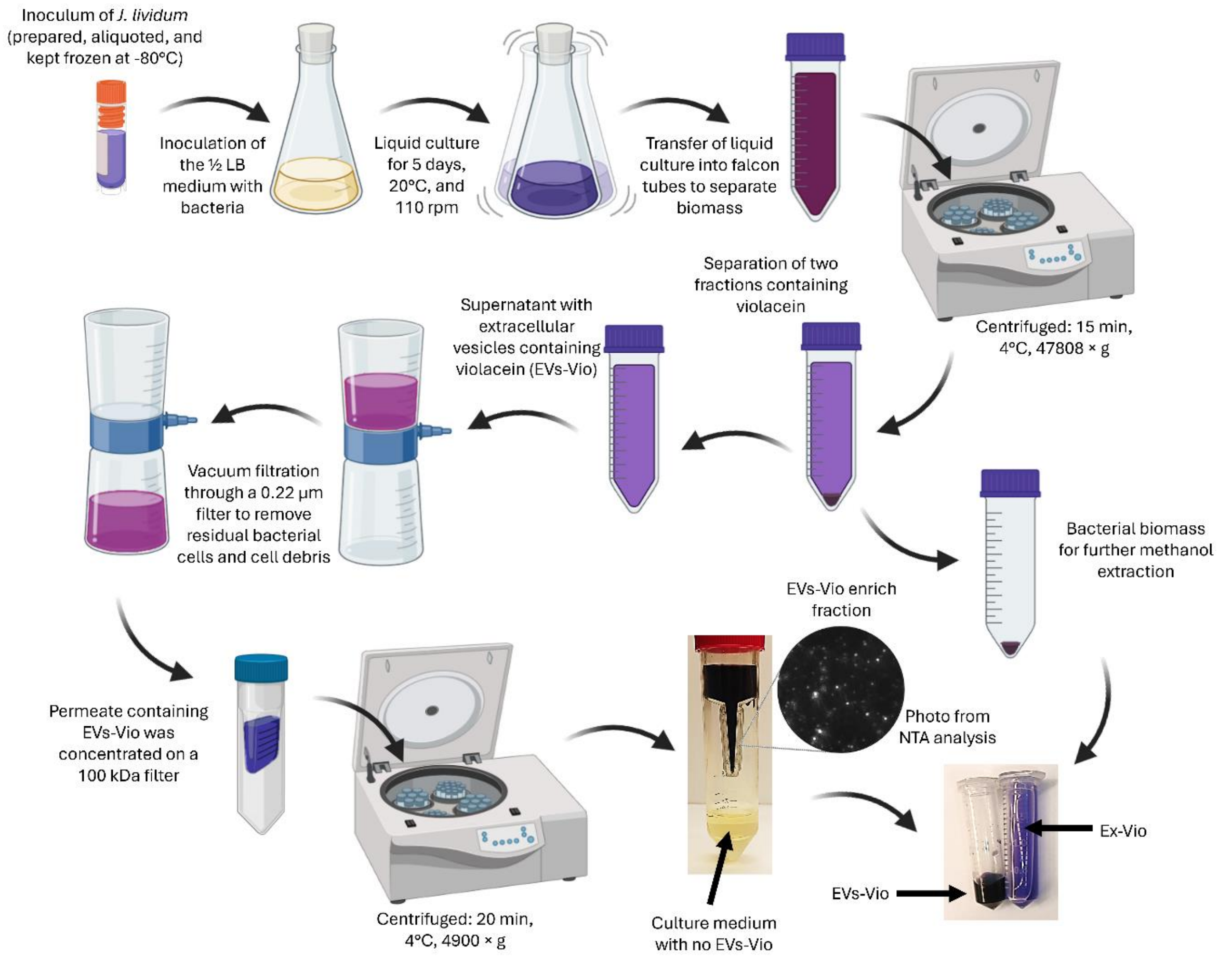

Fig. S1 Scheme for the purification of the extract and of the EVs from the culture of *J. lividum* (PCM 3520). This diagram was created in part with the help of BioRender (https://www.biorender.com/).

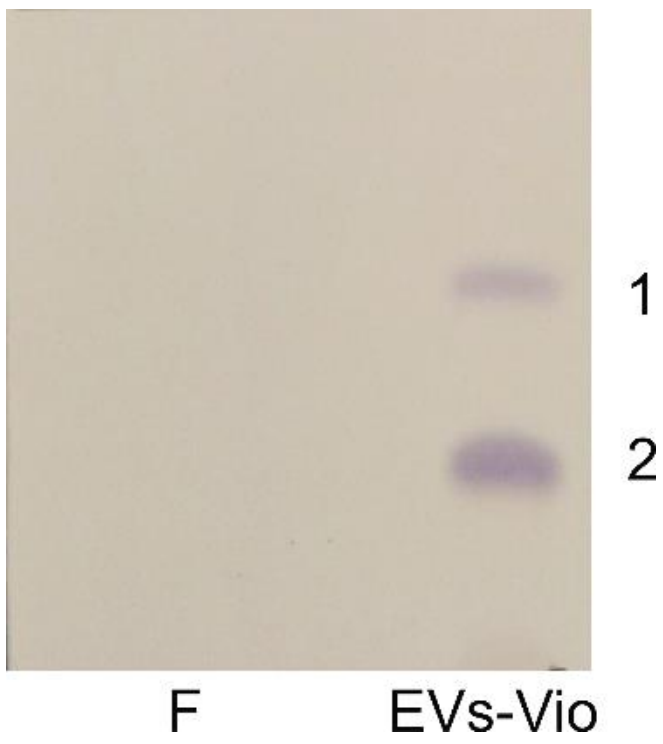

Fig. S2 Representative thin layer chromatography of the filtrate (F) and the *J. lividum* EVs containing violacein (EVs-Vio) obtained after filtration (cut-off of 100 kDa) of the culture medium.

Samples of EVs and the filtrate obtained after filtration of the culture medium on filters with a cut-off of 100 kDa were prepared by dilution in 100% methanol (50x). Thin layer chromatography (TLC) separation was performed using the solvent system acetone: chloroform: ammonia water in a ratio of 1:1:0.01 (MilliporeSigma, Burlington, VT, USA), on TLC silica gel 60 F254 (Merck, Darmstadt, Germany). After separation, the retention factor (Rf) was calculated using the formula:

$$Rf = \frac{Distance\ from\ start\ line\ to\ centre\ of\ spot}{The\ distance\ from\ the\ starting\ line\ to\ the\ front\ of\ the\ solvent}$$

After TLC separation, no additional contamination was observed at 254 and 365 nm. Under visible light, no dye was observed in the filtrate (Fig. S1). Spotting was observed only in the sample containing purified EVs with an Rf of (1) 0.58 and (2) 0.28. As the crude violacein is composed of violacein and deoxyviolacein differing by the presence of a polar hydroxyl group at the indole ring, the dye labelled (1) was interpreted as deoxyviolacein, while (2) was interpreted as violacein (as violacein binds more strongly to the polar solid phase).

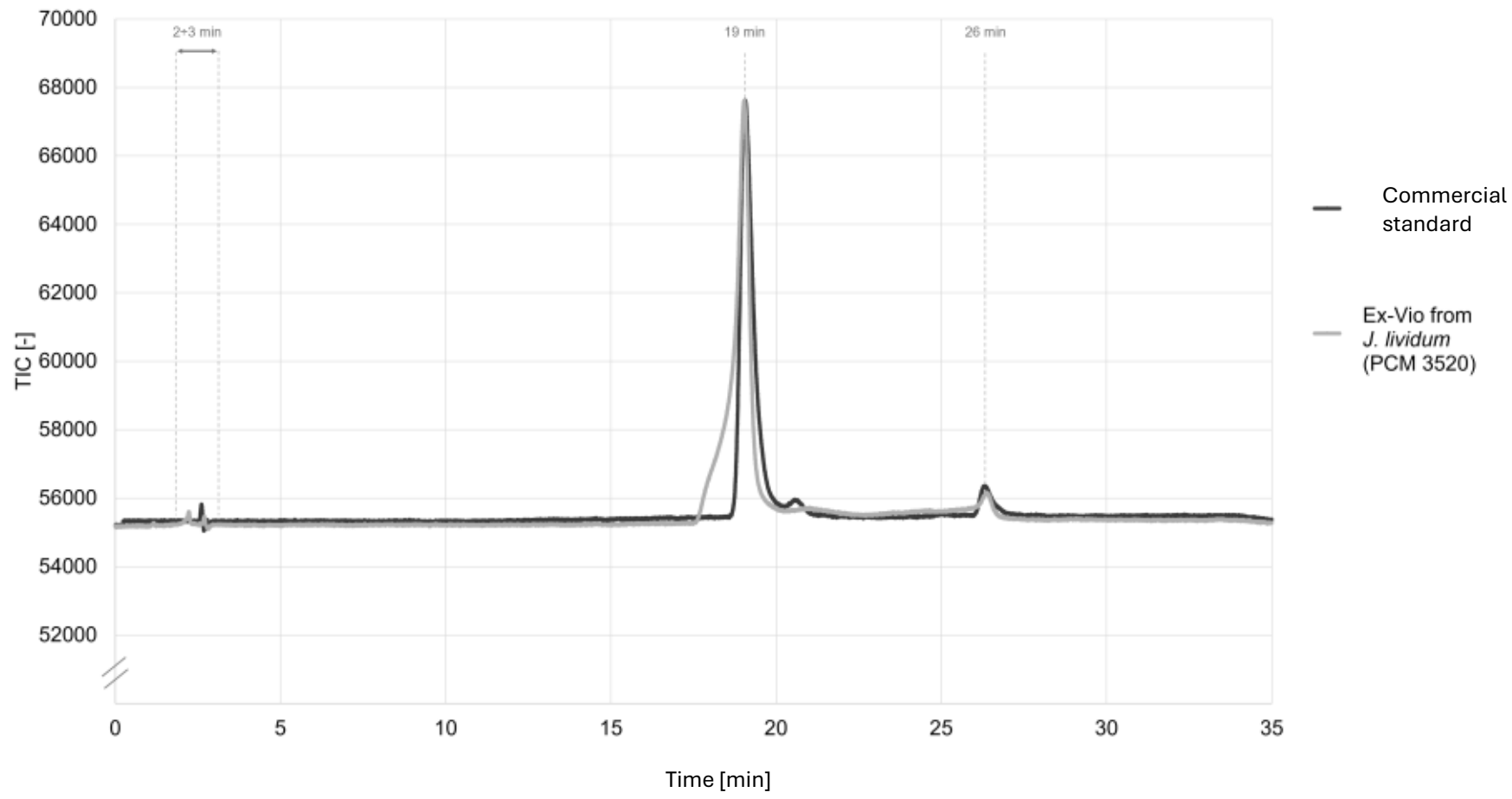

Fig. S3 Representative HPLC analysis of crude methanol extract of violacein from *J. lividum* (PCM 3520) vs. commercial standard of violacein extract from *J. lividum* (CAS No.: 548-54-9, MilliporeSigma, Burlington, VT, USA) dissolved in 100% methanol.

The HPLC analysis was performed using a C18 column as the stationary phase, and the mobile phase gradient composition between methanol (MilliporeSigma, Burlington, VT, USA) and water (MeOH:H_2O) was changed from an initial 50:50 (time 0.0 - 0.5 min) to 75:25. The proportion of MeOH mobile phase was increased linearly over the time 0.5 - 35 min. The flow rate was constant at 1 ml/min and detection was performed at 575 nm.

Chromatogram analysis of violacein from *J. lividum* (PCM 3520) extract indicates a purity of the dye of over 99%, including a violacein content of 97% and a deoxyviolacein content of 5.9%. Similarly, the purity of the commercial preparation measured by the same method was greater than 99%. In our opinion, the parameters of the preparation obtained in our laboratory are comparable to those of the commercial preparation.

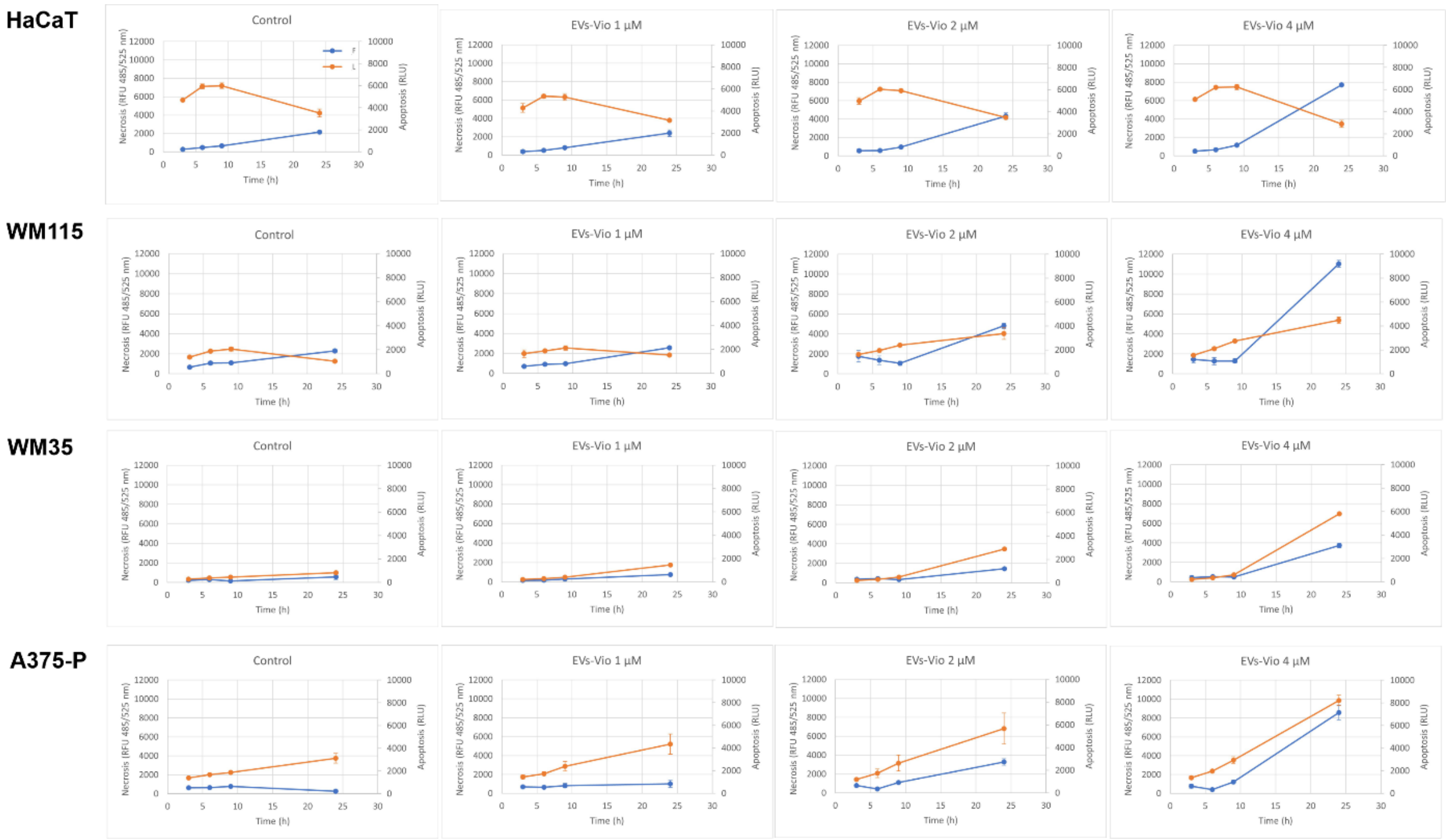

Fig. S4 Identification of apoptosis and necrosis processes in HaCaT and melanoma cells after treatment with 1, 2 and 4 µM violacein in the form of EVs. The test (the RealTime-Glow Annexin V Apoptosis and Necrosis Assay) compared the luminescence signal (associated with apoptosis) and fluorescence signal (associated with necrosis). Luminescence in relative light units (RLU, orange curve) and fluorescence in relative fluorescence units (RFU, blue curve) are plotted against the time of measurement. The assay was performed in three independent experiments.

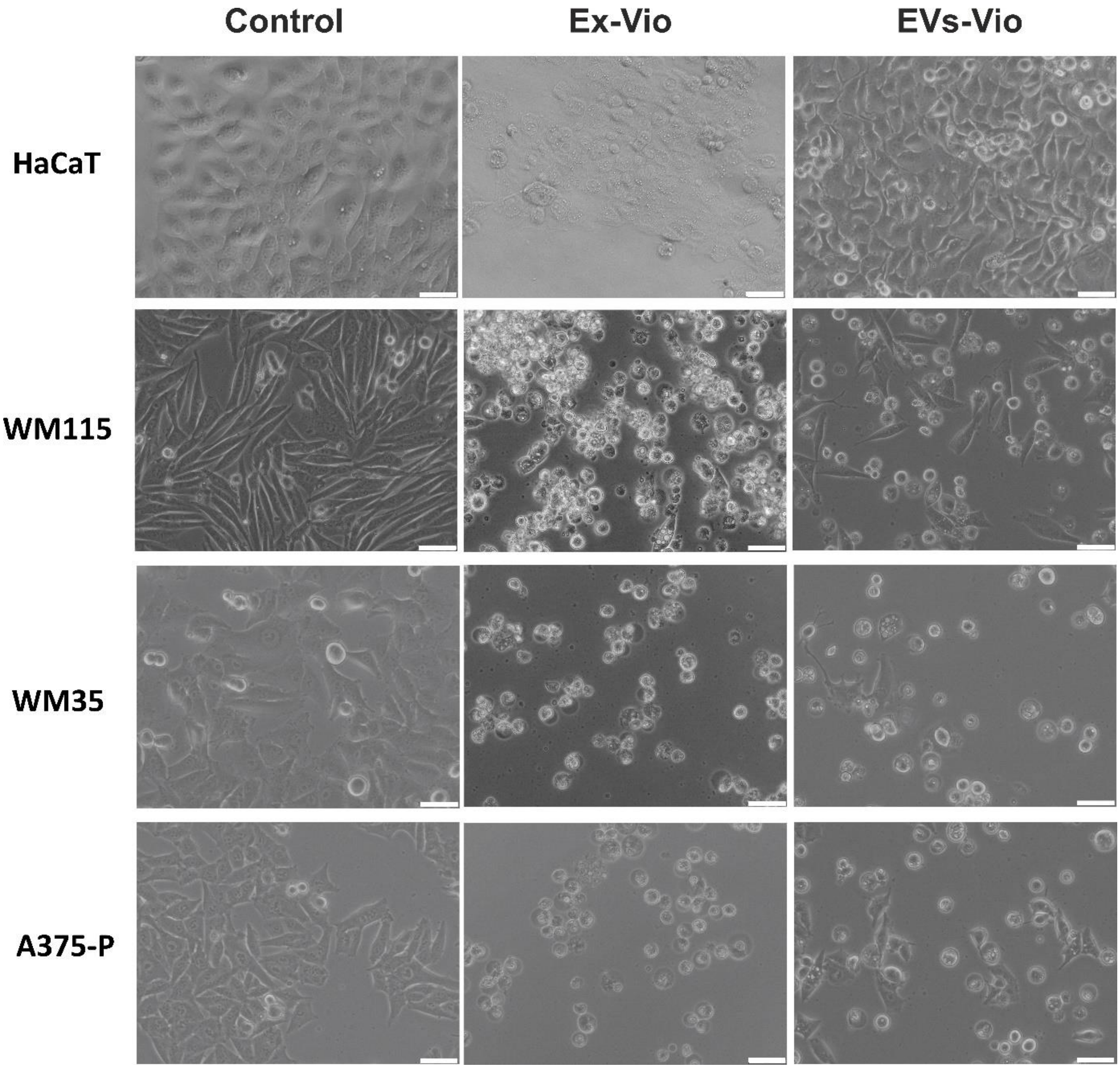

Fig. S5 In vitro morphological changes of HaCat and melanoma cells after 24 h of stimulation with EVs-Vio and Ex-Vio (at a concentration corresponding to 2 μM violacein). The pictures were taken at a magnification of 20×, and the scale bar indicates 50 μm.

Changes in cell morphology after Ex-Vio and EVs-Vio treatment indicate apoptosis (visible apoptotic bodies).
